# The Metaproteomics Initiative: a coordinated approach for propelling the functional characterization of microbiomes

**DOI:** 10.1186/s40168-021-01176-w

**Published:** 2021-12-20

**Authors:** Tim Van Den Bossche, Magnus Ø. Arntzen, Dörte Becher, Dirk Benndorf, Vincent G. H. Eijsink, Céline Henry, Pratik D. Jagtap, Nico Jehmlich, Catherine Juste, Benoit J. Kunath, Bart Mesuere, Thilo Muth, Phillip B. Pope, Jana Seifert, Alessandro Tanca, Sergio Uzzau, Paul Wilmes, Robert L. Hettich, Jean Armengaud

**Affiliations:** 1 VIB-UGent Center for Medical Biotechnology, VIB, 9000 Ghent, Belgium; 2 Department of Biomolecular Medicine, Faculty of Medicine and Health Sciences, Ghent University, 9000 Ghent, Belgium; 3 Faculty of Chemistry, Biotechnology and Food Science, NMBU-Norwegian University of Life Sciences, 1432 Ås, Norway; 4 Institute for Microbiology, Department for Microbial Proteomics, University of Greifswald, 17498 Greifswald, Germany; 5 Bioprocess Engineering, Otto von Guericke University, 39106 Magdeburg, Germany; 6 Bioprocess Engineering, Max Planck Institute for Dynamics of Complex Technical Systems, 39106 Magdeburg, Germany; 7 Microbiology, Anhalt University of Applied Sciences, 06354 Köthen, Germany; 8 Université Paris-Saclay, INRAE, AgroParisTech, Micalis Institute, 78350 Jouy-en-Josas, France; 9 Department of Biochemistry, Molecular Biology, and Biophysics, University of Minnesota, 6-155 Jackson Hall, 321 Church Street SE, Minneapolis, MN 55455 USA; 10 Helmholtz-Centre for Environmental Research GmbH-UFZ, Department of Molecular Systems Biology, Permoserstrasse 15, 04318 Leipzig, Germany; 11 Luxembourg Centre for Systems Biomedicine and Department of Life Sciences and Medicine, University of Luxembourg, Esch-sur-Alzette, Luxembourg; 12 Department of Applied Mathematics, Computer Science and Statistics, Ghent University, Ghent, Belgium; 13 Section eScience (S.3), Federal Institute for Materials Research and Testing, Berlin, Germany; 14 Faculty of Biosciences, NMBU - Norwegian University of Life Sciences, 1432 Ås, Norway; 15 HoLMiR - Hohenheim Center for Livestock Microbiome Research, University of Hohenheim, Leonore-Blosser-Reisen-Weg 3, 70599 Stuttgart, Germany; 16 Institute of Animal Science, University of Hohenheim, Emil-Wolff-Str. 6-10, 70599 Stuttgart, Germany; 17 Center for Research and Education on the Microbiota, Department of Biomedical Sciences, University of Sassari, Sassari, Italy; 18 Biosciences Division, Oak Ridge National Laboratory, Oak Ridge, TN USA; 19 Université Paris-Saclay, CEA, INRAE, Département Médicaments et Technologies pour la Santé (DMTS), SPI, 30200 Bagnols-sur-Cèze, France

**Keywords:** Microbiome, Metaproteomics, Multi-omics, Education, Interactions, Networking

## Abstract

Through connecting genomic and metabolic information, metaproteomics is an essential approach for understanding how microbiomes function in space and time. The international metaproteomics community is delighted to announce the launch of the Metaproteomics Initiative (www.metaproteomics.org), the goal of which is to promote dissemination of metaproteomics fundamentals, advancements, and applications through collaborative networking in microbiome research. The Initiative aims to be the central information hub and open meeting place where newcomers and experts interact to communicate, standardize, and accelerate experimental and bioinformatic methodologies in this field. We invite the entire microbiome community to join and discuss potential synergies at the interfaces with other disciplines, and to collectively promote innovative approaches to gain deeper insights into microbiome functions and dynamics.

**Video Abstract**.

**Supplementary Information:**

The online version contains supplementary material available at 10.1186/s40168-021-01176-w.

## Background

Microbial communities are major drivers of biogeochemical cycles, vital components of the biosphere including plants and animals, and key factors in human health. High-throughput genome sequencing technologies have revealed the immense diversity of microbes and the complexity of the ecological interactions within microbiomes [1]. In the past, deciphering the functioning of these complex microbial systems has been a daunting challenge, but is now becoming more approachable due to recent technological advances that allow diving much deeper into the functional biomolecular machinery. Metaproteomics is one such emerging approach that is beginning to dramatically advance our understanding of microbiome functioning through analyzing the spatio-temporal expression of microbial genes and dynamics within a microbial consortium.

Proteins are the workhorses of metabolism, homeostasis, cell division, transport of nutrients, cell-cell communication, protein synthesis, and the construction of cellular and extracellular structures. While the proteome defines the set of proteins in a single organism, the metaproteome comprises the complete suite of proteins from the variety of microorganisms present in a sample or ecosystem. As a result, a myriad of proteins, some of which may share relatively similar sequences, should be identified and quantified to acquire phenotypically relevant knowledge of the functioning of microbiomes [2]. Importantly, metaproteomic information complements the genetic potential and gene expression information obtained through metagenomics and metatranscriptomics, respectively, by identifying the temporal and spatial abundance of metabolic enzymes and other proteins. Metaproteomics is also highly complementary to metabolomics in explaining the phenotype of biological systems. Importantly, due to sequence variation among metabolic enzymes, metaproteomics data provides insight into “which organism is doing what” in a microbiome ecosystem and enables identification of specific members of the microbiome that are responsible for a change in abundance of a given metabolite. Thus, metaproteomics is pivotally positioned amongst other multi-omics approaches to enable direct genotype-phenotype linkages. In particular, it facilitates the connection between nucleotide information and metabolic outputs. Furthermore, the identification of structural proteins, biofilm protein components, and other extracellular enzymes sets metaproteomics apart from the other omics layers, resulting in unique insights into microbiome functioning. Thereby, it provides robust data for unraveling microbial networks within microbiomes and contributes unique information to fully understand the interactions with their environment [3, 4]. Metaproteomics can capture data across all domains of life, which is crucial for example to understand host-associated ecosystems in which proteins and peptides represent essential molecular currencies of exchange. Hence, metaproteomics “snapshots” at the host-symbiont interfaces are unique in mapping out microbial and host functions that are reciprocally related. Improvements in sample preparation, high-resolution peptide/protein separation and analysis, and refined strategies for data interpretation have propelled metaproteomics dramatically forward in the past decade [4–7]. Indeed, metaproteomics has already helped answer a number of research questions that otherwise would have remained elusive, primarily by functional assessment of microbial communities based on the identification and quantification of taxon-specific peptides [8]. For example, whether polyphenols accumulated in soils are microbially unavailable under anoxia was challenged with metaproteomics on laboratory-controlled reactors [9]; unusual pathways for carbon and energy use were highlighted in *Olavius algarvensis*, a gutless marine worm relying on its bacterial symbionts to cope with nutrient-poor environments [10]; and fecal metaproteomics of patients with metabolic syndrome revealed the level of gut inflammation and the subtle changes in bacterial composition and their metabolism [11].

## Introduction of the metaproteome initiative

While metagenomic and metatranscriptomic approaches share a common technical measurement platform that is fairly uniform and standardized, the experimental protocol for metaproteomics is much more broad and under active development. This presents challenges for assessing and comparing results and capabilities. In addition, metaproteomics currently benefits greatly by the sequencing, assembly, and annotation of metagenomes for accurate interpretation of the data. Thus, to propel metaproteomics to an unprecedented level of microbiome functional analyses requires an expanded and concerted stage of development where all possible synergies between analytical scientists, protein biochemists, bioinformaticians, statisticians, microbiologists, and other multi-omics specialists should be exploited. The current positive momentum of metaproteomics is particularly ripe for such a shift in gear. Importantly, the microbiome research community needs knowledge dissemination via improved educational resources so that community members are informed of the possibilities metaproteomics can offer and have an understanding of the realistic status of the field. Ideally, the microbiome and metaproteomics community should have easier access to the most recent updates of methodologies through specialized open platforms equipped with state-of-the-art instrumentation and strong expertise in data analysis. The full impact of active research to further improve metaproteomics in terms of methodology and data analysis will only be achieved if we improve its standards, educate the whole community, and foster new conceptual advances, thus facilitating the applicability of metaproteomics to solve a wide range of key biological questions. Clearly, metaproteomics has now matured sufficiently to be a viable component of experimental design in current and future microbiome research projects.

A growing community of researchers using metaproteomics had the opportunity to meet at several international symposia (Magdeburg, Germany in February 2016, Alghero, Italy, in June 2017, Leipzig, Germany in December 2018, an online edition in June 2021, and Luxembourg City, Luxembourg in September 2021) and launched specific actions such as training sessions and interlaboratory comparisons. Over the past year, members of this community have regularly discussed a variety of subjects, such as the need for a place where newcomers and experts can discuss and initiate new projects, the desire to forge metaproteomics as a critical methodology of choice for functional analyses, and the need to establish and disseminate the best options and standards for this methodology. This has enabled the development of a roadmap in order to assess the possibilities of tackling more complex projects, such as meta-omics projects and spatio-temporal studies of hundreds of differing conditions involving a large number of samples and replicates to be analyzed at increasing depth and throughput.

This international community, which at the time of writing brings together over 120 members from 48 research groups from 15 countries, has proposed a new initiative under the aegis of the European Proteomics Association (EuPA, https://eupa.org), the federation of European national proteomics societies, with a clear mission and vision statement: “the Metaproteomics Initiative promotes dissemination of metaproteomics fundamentals, advancements, and applications through collaborative networking in microbiome research. We aim to be the central information hub and open meeting place where newcomers and experts interact to communicate, standardize, and accelerate experimental and bioinformatic methodologies in this field”. This will be achieved initially through a dedicated website (https://metaproteomics.org) and Twitter account (@MetaP_Init), presentations and other educational resources, an online communication channel, inter-laboratory comparisons, and regular symposia. We are delighted to announce that as of February 2021 the Metaproteomics Initiative was officially launched and supported by EuPA. We are confident that this Initiative will benefit from an open structure, facilitating exchanges and interactions with other disciplines, and favouring additional synergies at the interfaces with other omics, data science, and microbiology. Through this commentary, we invite the entire microbiome community to join this Initiative and promote collective innovative and holistic approaches to understand how microbiomes function.

The formulation and execution of a community-driven, multi-lab comparison of established metaproteomic workflows, the “Critical Assessment of MetaProteome Investigation” (CAMPI) study, is the first tangible action of the Metaproteomics Initiative [12]. CAMPI has compared sample preparation, mass spectrometry analysis, and bioinformatics workflows for data analysis. This benchmarking effort has led to findings which have highlighted the importance of each step of the analytical pipeline. Such a benchmarking effort calls for discussion not only on the needs of standardized experimental procedures and data treatment workflows, but also specific guidelines in data reporting and data sharing. The CAMPI effort has also paved the way for subsequent inter-laboratory assays where advanced methodological questions will be tackled by the strength and multiple expertises of the whole community. In this regard, it is clear that the definition of appropriate standardized biological samples or benchmark datasets presents important tasks for the community.

Among the other major activities already initiated by the Metaproteomics Initiative are (i) an inaugural online symposium in June 2021, (ii) its promotion among the microbiome community by means of active communication channels, (iii) gathering tutorials and lectures on metaproteomics for offering simple and free access to high-standard educational resources, and (iv) establishment of a consensual framework to stimulate, initiate and coordinate open working groups conducting projects with defined objectives and outputs. To achieve its objectives, the Metaproteomics Initiative will favor the organization of training courses for newcomers and advanced users, further promote its vision in regions currently under-represented, regularly organize new CAMPI editions open to all volunteers, propose brainstorming sessions and hackathons for solving specific bottlenecks, arrange specific round-tables for improving guidelines, and recommending metrics for high-quality results, and establish a platform where scientists with key scientific questions can meet metaproteomics specialists for smooth and efficient collaboration.

As pointed out above, the benefits of a united and stronger metaproteomics community and its successful insertion at the crossroads of many disciplines are huge. Although under the EuPA umbrella, the Metaproteomics Initiative is not limited to a given geographical area and welcomes all positive ideas and feedback. As the puzzling complexity of microbiome samples has been an important driver of methodological improvements in meta-omic sciences, open challenges in metaproteomics are expected to further stimulate significant advancements in mass spectrometry-based proteome analysis and proteome bioinformatics [12, 13]. Due to the myriad of possible applications, metaproteomic approaches are increasingly used and the field is becoming attractive to young scientists. Indeed, a new generation of scientists trained in one or more of the interrelated areas of meta-omics, data science, modeling, and microbiology is emerging. Engaging, inspiring, and educating early-career researchers are undoubtedly a rewarding prospect for the microbiome community.

## Conclusions

Ambitious long-term plans such as the Human Genome Project, the European Green Deal, and the Human Brain Project have pushed the boundaries of science. The multidisciplinary giant Tara Oceans project aimed at deciphering the complexity of ocean life by means of planetary exploration sampling and huge metagenomics efforts is an emblematic model for environmental microbiology [14]. The International Human Microbiome Standards Project is also a pivotal project for improving data quality and optimizing omics comparability in the human microbiome field. Such long-range frameworks offer challenging objectives for many researchers, cutting-edge research infrastructure to advance methodologies and knowledge, and a visibility that is attractive for stakeholders, media, and the public. The alliance of the Metaproteomics Initiative and other microbiome disciplines could become the cornerstone of such a long-term, cutting-edge project on the comprehensive molecular analysis of microbiome ecosystem functioning and adaptation. Through its unique positioning, we are confident that the Metaproteomics Initiative will contribute to the best possible returns on investment for microbiome research.

## Data Availability

Not applicable.
